# Efficacy of fish oil supplementation on metabolic dysfunction-associated steatotic liver disease: a meta-analysis

**DOI:** 10.3389/fnut.2025.1524830

**Published:** 2025-01-24

**Authors:** Like Zhou, Dongmei Sun, Houqiao Bai

**Affiliations:** Department of Gastroenterology, Weihai Maternal and Child Health Hospital, Weihai, China

**Keywords:** MASLD, fish oil, meta-analysis, NAFLD, NASH

## Abstract

**Objective:**

Globally, the occurrence of Metabolic dysfunction-associated steatotic liver disease (MASLD) is on a steady rise. Fish oil has anti-inflammatory effects and can improve lipid metabolism. The article aims to assess the impact of fish oil supplementation on MASLD.

**Methods:**

We conducted a systematic search of Cochrane, Embase, PubMed, and Web of Science up to September 31, 2024, for randomized control trials (RCTs). The risk of bias of the included RCTs was evaluated using the Cochrane Collaboration’s tool. Outcomes measured were aspects of liver injury, lipid profile, insulin resistance, anthropometric measurements, and more.

**Results:**

Seven randomized controlled trials (RCTs) involving 439 participants were incorporated into the analysis. In general, the risk of bias in these RCTs was either low or not clearly defined. Pooled analysis showed that triglycerides [TG, pooled standard mean difference (SMD): −0.40 (95% CI: −0.58 to −0.21)], aspartate transaminase [AST, SMD: −0.29 (95% CI: −0.48 to −0.10)], HOMA-IR [SMD: −2.06 (95% CI: −3.36 to −0.49)] and waist circumference [Waist-C, SMD: −0.31 (95% CI: −0.54 to −0.08)] were significantly improved. But showed no significant benefits on alanine transaminase [ALT, SMD: −0.15 (95% CI: −0.45 to 0.15)], gamma-glutamyl transpeptidase [GGT, SMD: −0.07 (95% CI: −0.26 to 0.12)], body mass index [BMI, SMD: 0.16 (95% CI: −0.34 to 0.02)], high-density lipoprotein cholesterol [HDL, SMD: 0.02 (95% CI: −0.18 to 0.22)], low-density lipoprotein cholesterol [LDL, SMD: −0.01 (95% CI: −0.20 to 0.18)], Total Cholesterol [TC, SMD: −0.34 (95% CI: −0.70 to 0.01)] and so on.

**Conclusion:**

The current evidence supports the fish oil supplementation in improving MASLD. Fish oil supplementation may also regulate blood lipids and improve glucose metabolism disorders.

**Systematic review registration:**

https://www.crd.york.ac.uk/PROSPERO/#myprospero, identifier CRD42024513246.

## 1 Introduction

Metabolic dysfunction-associated steatotic liver disease (MASLD) represents a significant chronic liver condition, spanning various clinical and pathological manifestations such as fatty degeneration, steatohepatitis, fibrosis, cirrhosis, and hepatocellular carcinoma (1). Up to 30% of adults were affected by MASLD in Western, which implied the obesity epidemic (2). Due to shifting lifestyles and dietary habits, the prevalence of MASLD in China has surged to 25%. Despite being asymptomatic in the early stages, MASLD is positively correlated with the risks of cardiovascular disease (CVDs) and type 2 diabetes (T2DM), as evidenced by substantial evidence (3, 4). Calorie restriction and exercise remain the primary treatments for reducing visceral obesity and liver steatosis (5). Evidence from randomized controlled trials clearly indicates that most patients find it difficult to maintain weight loss (6). Tofogliflozin, Sitagliptin, Semaglutide, Pioglitazone, and Ursodeoxycholic Acid have demonstrated efficacy in improving inflammation, insulin resistance, liver function, and histological features of MASLD (7). Treatment of MASLD remains challenging for the scientific community despite numerous clinical trials, with no approved treatments currently available.

Docosapentaenoic acid (DPA) and docosahexaenoic acid (DHA) are constituents of fish oil, categorized as Omega-3 polyunsaturated fatty acids (n-3 PUFAs). These compounds have demonstrated efficacy in treating cardiovascular disease (CVD) by reducing triglycerides and regulating inflammation (8). *ω*-3 PUFAs may additionally enhance the observed decrease in total body fat during weight loss induced by diet (9). Studies on total parenteral nutrition have confirmed that prolonged dietary deficiency in *ω*-3 PUFAs can result in liver steatosis (10–12). Evidence from animal (10, 13) and human (14) studies suggests that *ω*-3 PUFA dietary supplements may prevent MASLD or reduce liver fat, independent of weight loss (10, 13, 14). ω-3 PUFAs effectively reduce abnormal triglyceride (TAG) levels (15–17). Lipidomics studies have revealed a significant correlation between a high liver N-6:N-3 ratio and MASLD severity (18). Several studies indicate that incorporating n-3 PUFAs into a low-fat diet can decrease steatosis and enhance liver enzymes and metabolic parameters (14, 19). Studies have demonstrated that MASLD patients exhibit significantly elevated levels of n-3 PUFAs, particularly DHA, in their blood compared to healthy subjects. Fish oil supplementation has been found to significantly improve liver function and lipid metabolism in MASLD patients (1, 20).

Numerous clinical trials and studies are investigating the effectiveness of fish oil supplementation in treating MASLD. Nevertheless, the most recent clinical data has not been included in meta-analyses for data aggregation, leading to insufficient evidence-based medicine to support this intervention. Therefore, a meta-analysis was conducted on the supplementation of fish oil in patients with MASLD. Our aim is to evaluate the effects of fish oil on liver injury, lipid profile, insulin resistance, anthropometric measurements, and other relevant parameters.

## 2 Materials and methods

### 2.1 Data sources and literature search strategy

This evidence-based analysis followed the PRISMA (Preferred Reporting Items for Systematic Reviews and Meta-Analysis) 2020 statement (21) and was prospectively registered in PROSPERO (CRD42024513246). We systematically searched the Cochrane, Embase, PubMed, and Web of Science databases up to 31 September, 2024. The search keywords: fish oils, Metabolic dysfunction-associated steatotic liver disease. The search strategy was ((“Fish Oils”[Mesh]) OR ((((((Oils, Fish) OR (Fish Oil)) OR (Oil, Fish)) OR (Fish Liver Oils)) OR (Liver Oils, Fish)) OR (Oils, Fish Liver))) AND ((“Non-alcoholic Fatty Liver Disease”[Mesh]) OR (((((((((((((Non alcoholic Fatty Liver Disease) OR (MASLD)) OR (Nonalcoholic Fatty Liver Disease)) OR (Fatty Liver, Nonalcoholic)) OR (Fatty Livers, Nonalcoholic)) OR (Liver, Nonalcoholic Fatty)) OR (Livers, Nonalcoholic Fatty)) OR (Nonalcoholic Fatty Liver)) OR (Nonalcoholic Fatty Livers)) OR (Nonalcoholic Steatohepatitis)) OR (Nonalcoholic Steatohepatitides)) OR (Steatohepatitides, Nonalcoholic)) OR (Steatohepatitis, Nonalcoholic))). The search results from four databases are presented in Supplementary Table S1. This trial had no language or geographical restrictions.

### 2.2 Study selection

Inclusion criteria were as follows: (1) The study design is RCT; (2) Participants diagnosed with MASLD; (3) Intervention group received fish oil supplementation, while the control group received placebo or other treatments. Title and abstract screening for eligibility was independently conducted by two authors (L.K.Z and D.M.S). Disagreements were resolved by consulting a senior author (H.Q.B). Reviews, letters, editorial comments, case reports, conference abstracts, non-human studies, unpublished articles, those with incomplete data, and non-English articles were excluded.

### 2.3 Data extraction

Two authors (L.K.Z and D.M.S) independently screened literature and extracted data from the included trials, including the first author’s last name, number of participants, publication year, country, and outcome data for both intervention and control groups. Any disagreements were resolved by a third investigator (H.Q.B.) for a final decision. The outcomes focused on were: (1) biochemical markers, including serum markers of liver injury (ALT, AST, GGT and CK18-M30) and lipid profiles (TC, TG, HDL and LDL), adiponectin, and UA; (2) insulin, HOMA-IR and FBS; and (3) anthropometric parameters, such as obesity estimated by BMI, waist circumference, hip circumference, and WHR.

### 2.4 Risk of bias assessment

**Figure 1:**
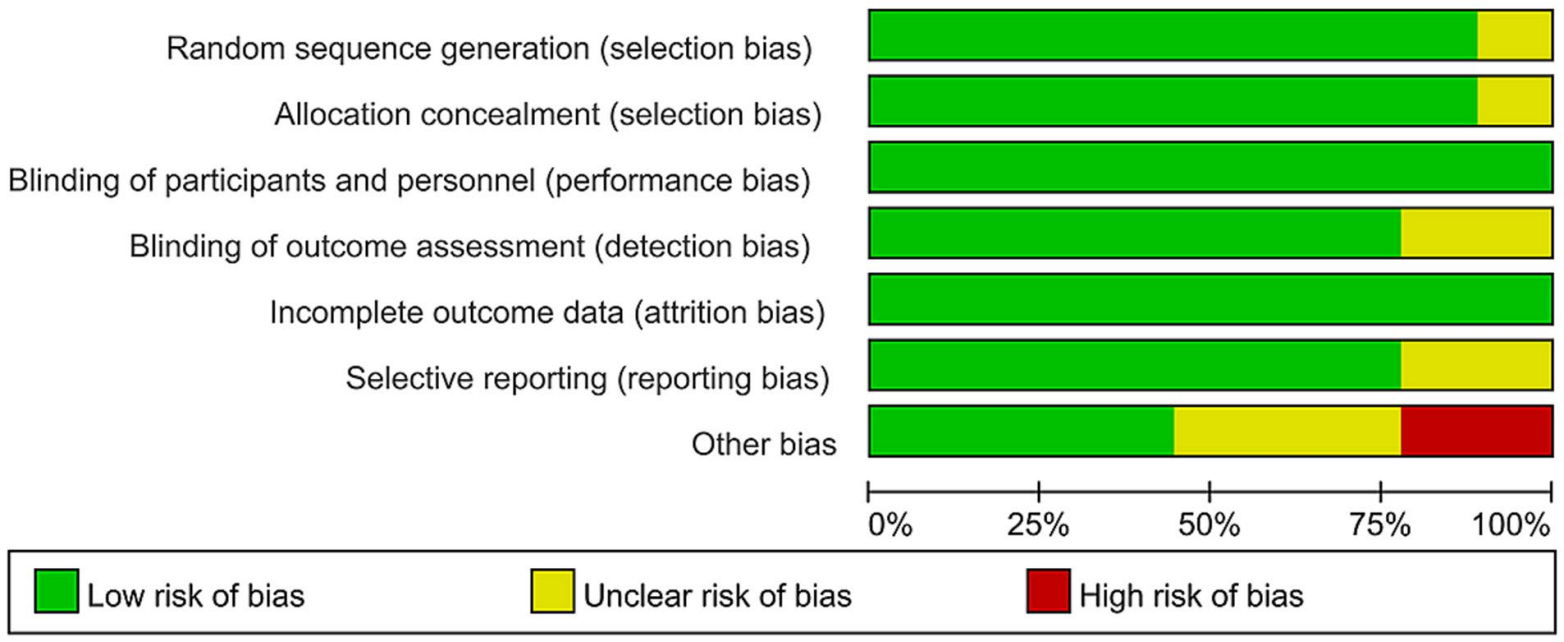
Risk of bias graph of included studies.

**Figure 2:**
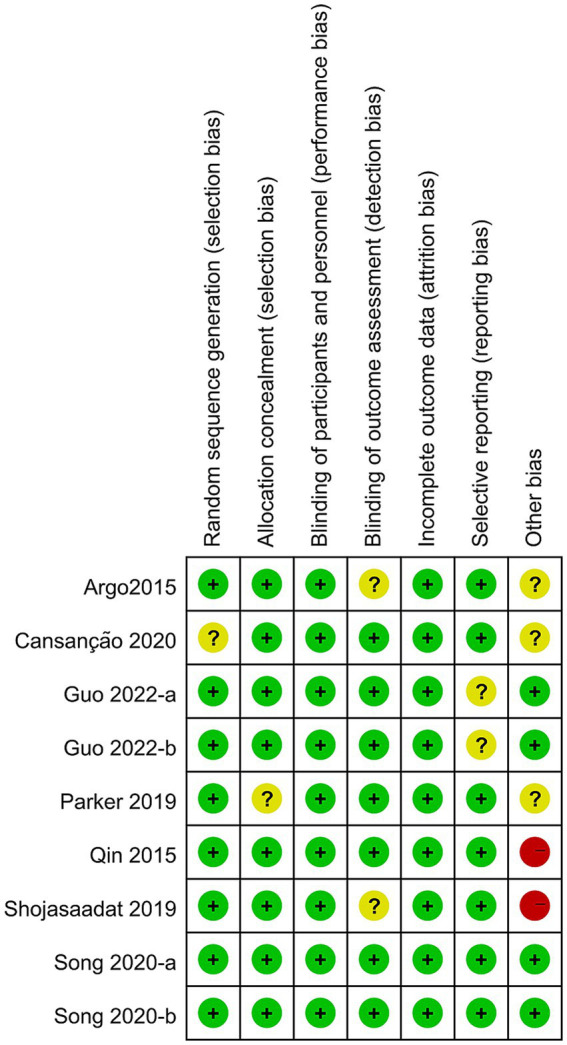
Risk of bias summary of included studies.

Two authors (L.K.Z and D.M.S) used tool (22) to assess the risk of bias in the included studies, with the third author (H.Q.B) responsible for confirming the judgment results. RCTs were assessed for high, low, or unclear risk of bias in six domains: randomization method, allocation concealment, blinding, completeness of results data, selective reporting of results, and other sources of bias. If a study did not provide data, it was rated as having an unclear risk of selective reporting bias (see Figures 1, 2).

### 2.5 Methodological quality evaluation

The methodological quality assessment was based on JADAD score (23), including the following: sequence generation, allocation concealment, blinding, withdrawals and drop outs, and randomization efficacy. The evaluation process was independently performed by two of the authors (L.K.Z and D.M.S). Disagreement was resolved by third author ((H.Q.B; Supplementary Table S2).

### 2.6 Statistical analysis

All analyses were conducted using Review Manager software version 5.4 (Nordic Cochrane Center, the Cochrane Collaboration, 2020). We anticipated clinical heterogeneity, so we chose a random-effects model. Dichotomous variables are presented as risk ratios (RR) with their 95% confidence intervals (95% CI). Continuous variables are expressed as standardized mean differences (SMD) with their 95% CI. Statistical heterogeneity between studies was assessed by calculating the I^2^ statistic. An I^2^ value greater than 50% was considered to indicate significant heterogeneity (24), a random-effect model was used to estimate the combined SMD when significant heterogeneity was detected (I^2^ > 50%). Otherwise, the fixed-effect model was applied. A *p*-value less than 0.05 was considered statistically significant. To assess the impact of individual studies on the pooled outcomes exhibiting notable heterogeneity, we further conducted one-way sensitivity analyses. The existence of publication bias was visually assessed using funnel plots generated in Review Manager 5.3 (Cochrane Collaboration, Oxford, United Kingdom). Additionally, Egger’s regression tests (25) were conducted in Stata 12.0 (Stata Corp, College Station, TX, United States) to further evaluate potential bias in outcomes with at least 10 included studies. A *p*-value less than 0.05 was deemed statistically significant, indicating the presence of publication bias. Our study conducts subgroup analysis based on the dosage of fish oil and the duration of intervention to explore the stability of the results and potential sources of heterogeneity.

## 3 Results

### 3.1 Characteristics of included studies

**Figure 3:**
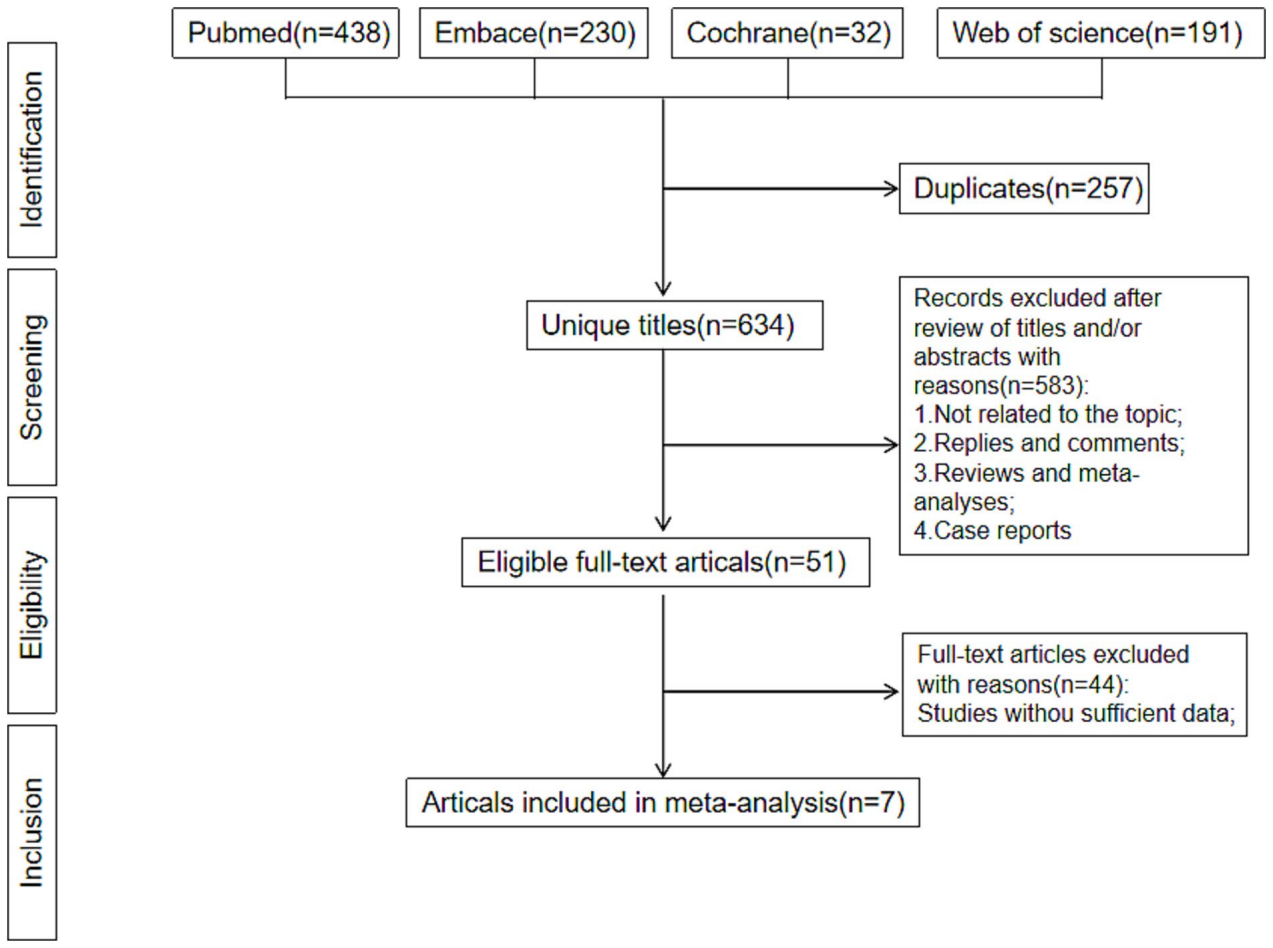
Flowchart of the systematic search and selection process.

After excluding duplicate literature, 623 literature identified in our research. Figure 3 shows the Preferred Reporting Items for Systematic Reviews and Meta-Analyses flowchart. After screening of titles and abstracts, 616 records were excluded. Seven RCTs, comprising 439 participants, were included after reviewing the full texts. Of these articles, there were 340 males and 99 females, with a minimum sample size of 34 and a maximum sample size of 74, a minimum mean age of 33.6 years and a maximum of 60.88 years, and a minimum mean body mass index of 26.0 and a maximum of 33.3. The study lasted for a minimum of 12 weeks and a maximum of 1 year. The characteristics of the included RCTs (26–32) were presented in Table 1.

**Table 1:** Baseline characteristics of include studies.

Authors	Study period	Country	Study design	Patients (n)	BMI	Age (mean/median)	Male	Time of duration
				Intervention/control	Intervention/control	Intervention/control	Intervention/control
Argo, 2015	2007-2010	USA	RCT	17/17	31.6/33.3	46.4/47.2	6/7	1 year
Qin, 2015	2012-2013	China	RCT	36/34	26.4/26.0	57/55	26/25	3 months
Parker, 2019	2011-2013	Australia	RCT	25/25	27.8/28.0	33.6/34.7	25/25	12 weeks
Shojasaadat, 2019	2016-2017	Iran	RCT	35/34	31.48/30.65	41.77/42.35	18/20	12 weeks
Song, 2020-a	2018-2019	China	RCT	21/21	29.43/27.92	46/47	19/18	12 weeks
Song, 2020-b	2018-2019	China	RCT	17/21	27.80/27.92	44/47	16/18	12 weeks
Cansanção, 2020	2018-2020	Brazil	RCT	13/11	30.77/31.82	60.54/60.88	8/9	6 months
Guo, 2022-a	2019-2021	China	RCT	37/37	27.6/26.7	54.7/56.3	22/19	3 months
Guo, 2022-b	2019-2021	China	RCT	37/37	26.2/26.7	56.6/56.3	20/19	3 months

### 3.2 Effects of fish oil on serum markers of liver injury

**Figure 4:**
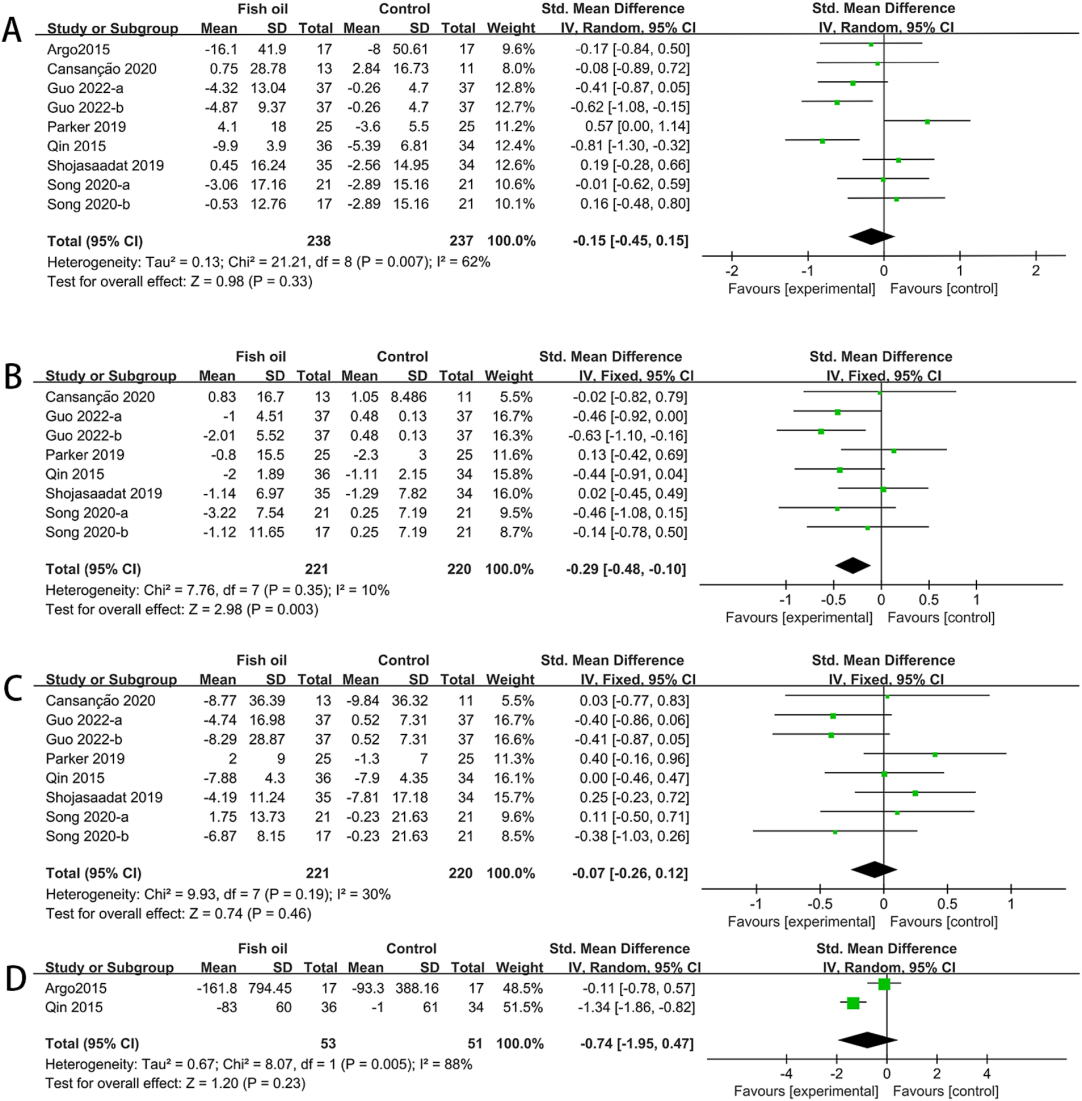
Forest plots of effects of fish oil on serum markers of liver injury: **(A)** ALT; **(B)** AST; **(C)** GGT; **(D)** CK18-M30.

The analysis involved data from 7 RCTs for AST (26–32), 6 for ALT and GGT (27–32), and 2 for CK18-M30 (26, 27). Significant statistical heterogeneity was observed among studies for AST (I^2^ = 62%, *p* = 0.007) and CK18-M30 (I^2^ = 88%, *p* = 0.005). The meta-analysis revealed that compared to the control group, the fish oil group exhibited a significant improvement in AST (SMD: −0.29, 95% CI: −0.48 to −0.10), whereas no significant differences were observed in ALT (SMD: −0.15, 95% CI: −0.45 to 0.15), GGT (SMD: −0.07, 95% CI: −0.26 to 0.12), or CK18-M30 (SMD: −0.74, 95% CI: −1.95 to 0.47; see Figure 4).

### 3.3 Effect of fish oil on serum lipid profiles

**Figure 5:**
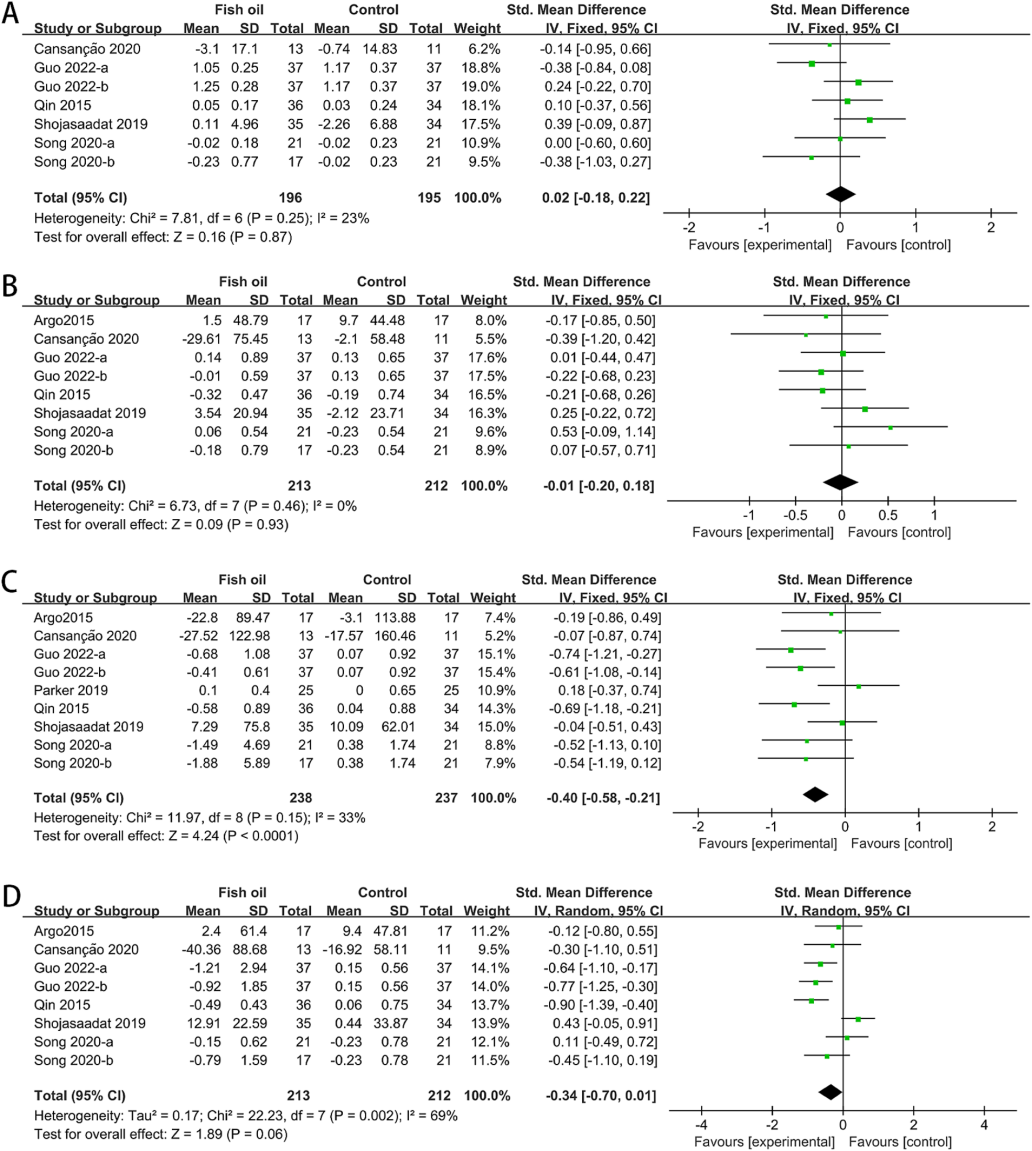
Forest plots of effect of fish oil on serum lipid profiles: **(A)** HDL-c; **(B)** LDL-c; **(C)** TG; **(D)** total cholesterol.

The meta-analysis included data from five RCTs for HDL (27, 29–32), six for LDL (26, 27, 29–32) and total cholesterol (TC) (26, 27, 29–32), and seven for triglycerides (TG) (26–32). Significant heterogeneity was observed among studies for total cholesterol (TC: I^2^ = 69%, *p* = 0.002). The fish oil group exhibited a significant improvement in triglycerides (SMD: −0.40, 95% CI: −0.58 to −0.21). However, no significant changes were found in HDL (SMD: 0.02, 95% CI: −0.18 to 0.22), LDL (SMD: −0.01, 95% CI: −0.20 to 0.18), or TC (SMD: −0.34, 95% CI: −0.70 to 0.01; see Figure 5).

### 3.4 Effect of fish oil on fasting blood sugar, insulin and homeostatic model assessment for insulin resistance

**Figure 6:**
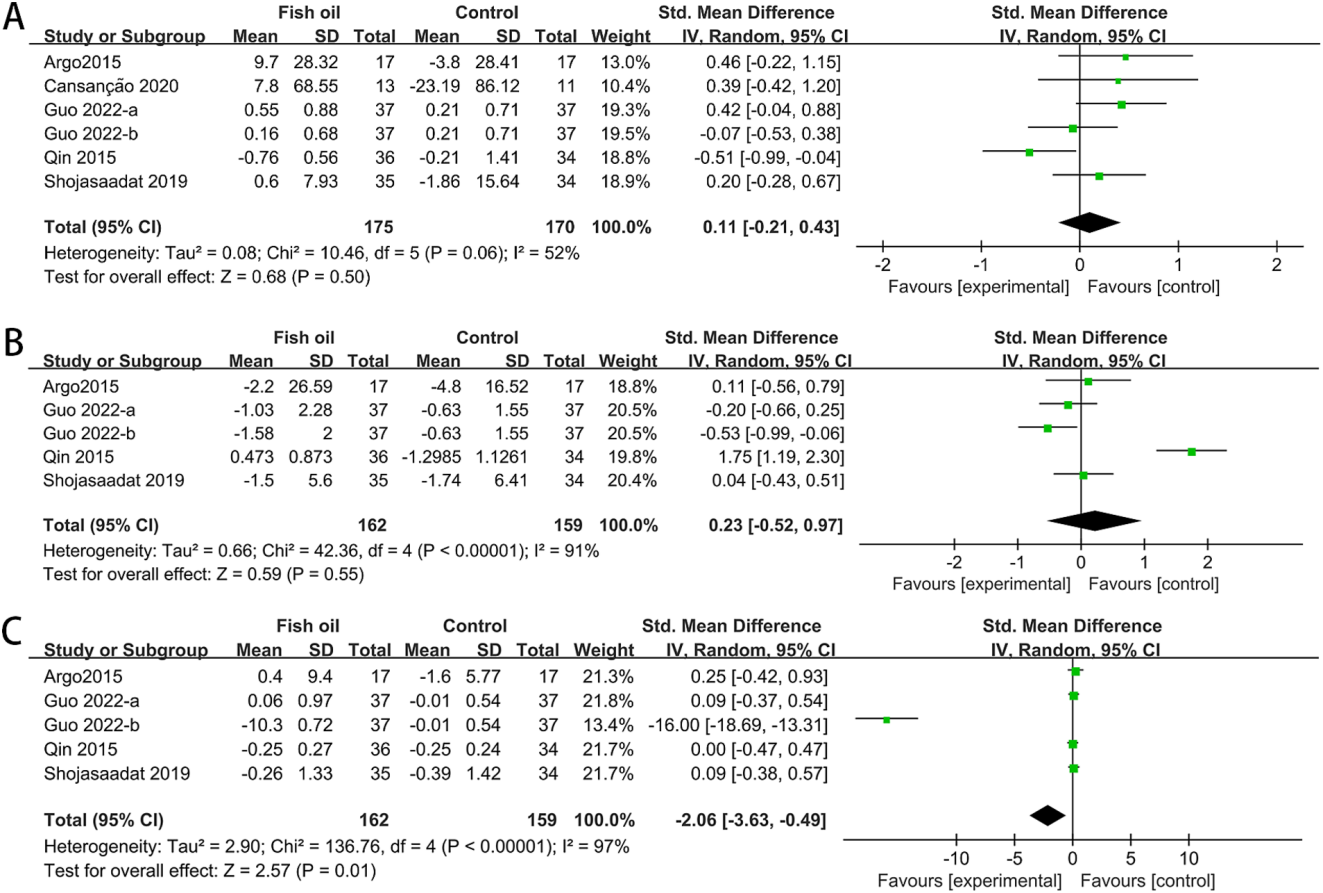
Forest plots of effect of fish oil on fasting blood sugar, insulin and homeostatic model assessment for insulin resistance: **(A)** FBS; **(B)** insulin; **(C)** HOMA-IR.

The meta-analysis included data from five for FBS (26, 27, 29, 30, 32), four for insulin and HOMA-IR values (26, 27, 29, 32). Significant heterogeneity was observed among studies for FBS (I^2^ = 52%, *p* = 0.06), insulin (I^2^ = 91%, *p* < 0.00001), and HOMA-IR (I^2^ = 97%, *p* < 0.00001). The meta-analysis showed a significant improvement in HOMA-IR with fish oil supplementation, no significant change in insulin levels, and a significant increase in FBS. The combined SMD for FBS was 0.08 (95% CI: −0.13 to 0.30), the combined SMD for insulin was 0.23 (95% CI, −0.52 to 0.97), and the SMD for HOMA-IR was −2.06 (95% CI, −3.36 to −0.49; see Figure 6).

### 3.5 Effect of fish oil on anthropometric measurements

**Figure 7:**
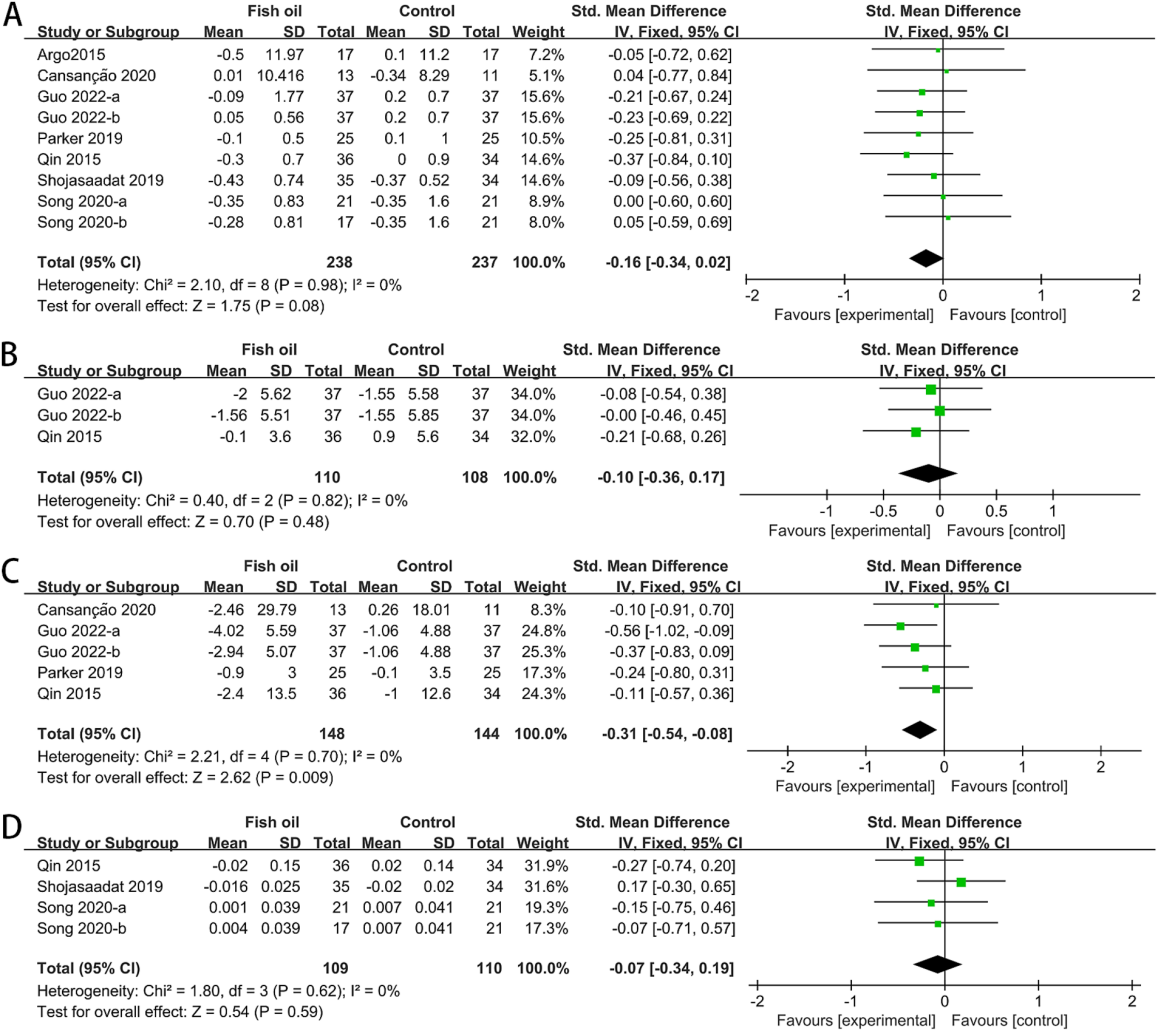
Forest plots of effect of fish oil on anthropometric measurements: **(A)** BMI; **(B)** Hip-C; **(C)** Waist-C; **(D)** WHR.

The analysis included data from seven RCTs for BMI (26–32), 2 for hip circumference (Hip-C) (27, 32), four for waist circumference (Waist-C) (27, 28, 30, 32), and three for waist-to-hip ratio (WHR) (27, 29, 31). No statistical heterogeneity was observed among the studies. The meta-analysis revealed a significant improvement in Waist-C (SMD: −0.31, 95% CI: −0.54 to −0.08) in the fish oil group. However, there were no significant improvements in BMI (SMD: 0.16, 95% CI: −0.34 to 0.02), Hip-C (SMD: −0.10, 95% CI: −0.36 to 0.17), or WHR (SMD: −0.07, 95% CI: −0.34 to 0.19; see Figure 7).

### 3.6 Effect of fish oil on adiponectin, UA and TNF-α

**Figure 8:**
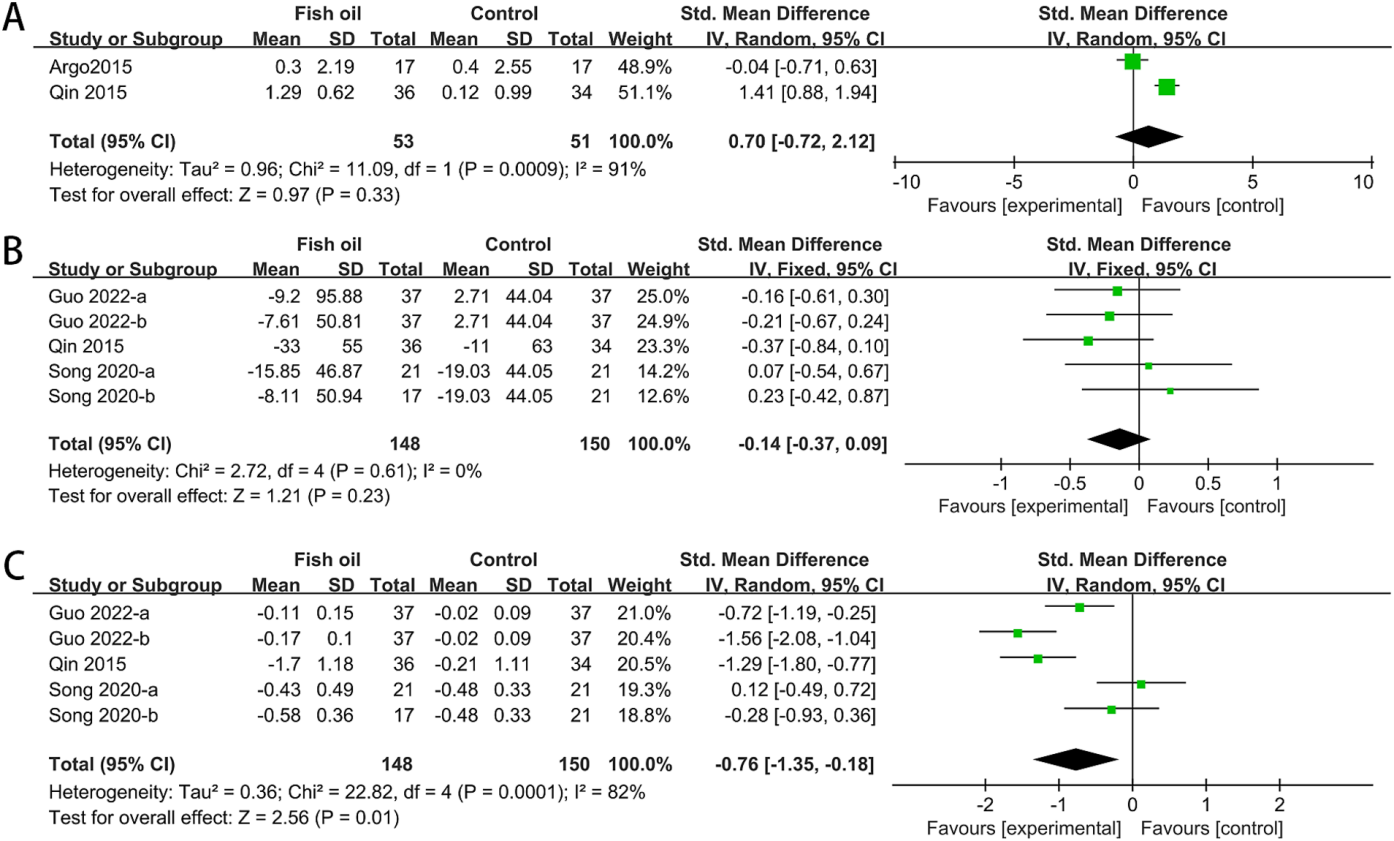
Forest plots of effect of fish oil on adiponectin, UA and TNF-α: **(A)** adiponectin; **(B)** UA; **(C)** TNF-α.

The analysis included data from two RCTs for adiponectin (26, 27), three for uric acid (UA) (27, 31, 32), and three for tumor necrosis factor-alpha (TNF-α) (27, 31, 32). Heterogeneity was observed among studies for adiponectin (I^2^ = 91%, *p* = 0.0009) and TNF-α (I^2^ = 82%, *p* = 0.0001). The meta-analysis revealed a significant improvement in TNF-α levels in the fish oil group compared to the control group (SMD: −0.76, 95% CI: −1.35 to −0.18). However, there were no significant changes in adiponectin (SMD: 0.70, 95% CI: −0.72 to 2.12) or UA (SMD: −0.14, 95% CI: −0.37 to 0.09; see Figure 8).

### 3.7 Publication bias

**Figure 9:**
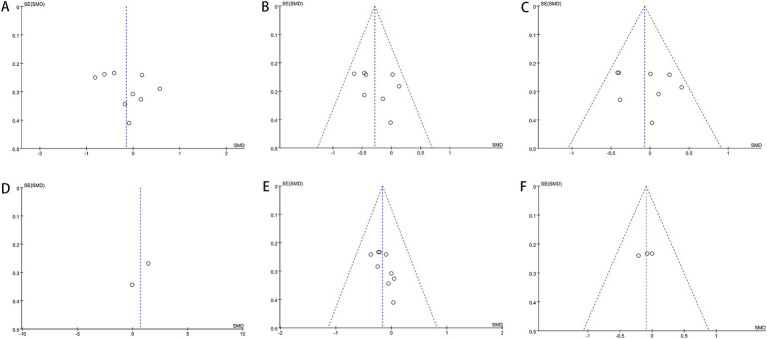
Funnel plots of **(A)** ALT, **(B)** AST, **(C)** GGT, **(D)** adiponectin, **(E)** BMI and **(F)** Hip-C.

**Figure 10:**
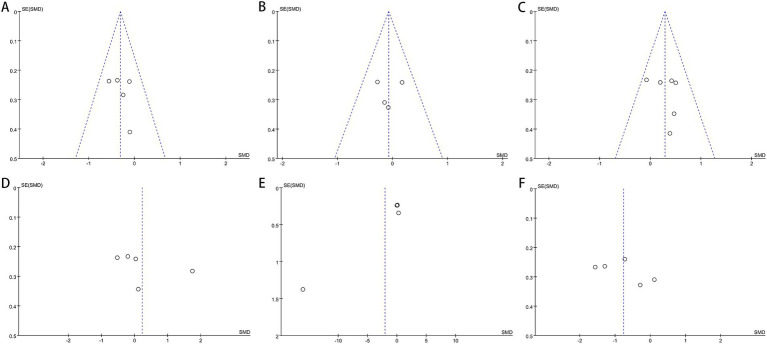
Funnel plots of **(A)** Waist-C, **(B)** WHR, **(C)** FBS, **(D)** insulin, **(E)** HOMA-IR and **(F)** TNF-*α*.

**Figure 11:**
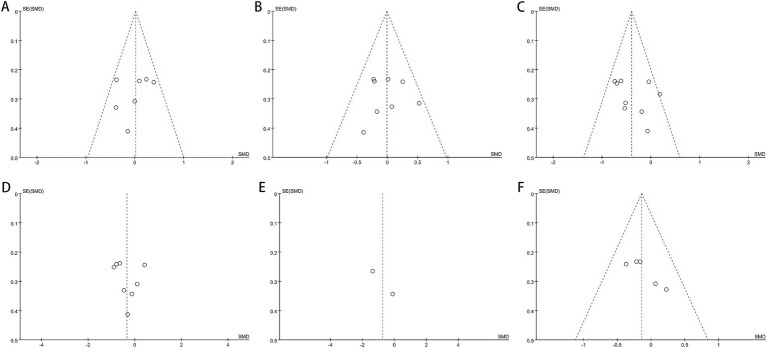
Funnel plots of **(A)** HDL-c, **(B)** LDL-c, **(C)** TG, **(D)** total cholesterol, **(E)** CK18-M30 and **(F)** UA.

Our investigation found that Egger’s test results indicated no publication bias for certain outcomes: AST (*p* = 0.302), ALT (*p* = 0.358), GGT (*p* = 0.638), HDL (*p* = 0.452), LDL (*p* = 0.964), TG (*p* = 0.387), TC (*p* = 0.616), Hip-C (*p* = 0.236), Waist-C (*p* = 0.475), WHR (*p* = 0.864), insulin (*p* = 0.431), FBS (*p* = 0.449), TNF-*α* (*p* = 0.303), and UA (*p* = 0.051). However, publication bias was suggested by the test for BMI (*p* = 0.034) and HOMA-IR (*p* = 0.024). Funnel plots also revealed publication bias in ALT, BMI, FBS, insulin, and HOMA-IR. Figures 9–11 demonstrate the visual assessment of funnel plots.

### 3.8 Sensitivity analysis

**Figure 12:**
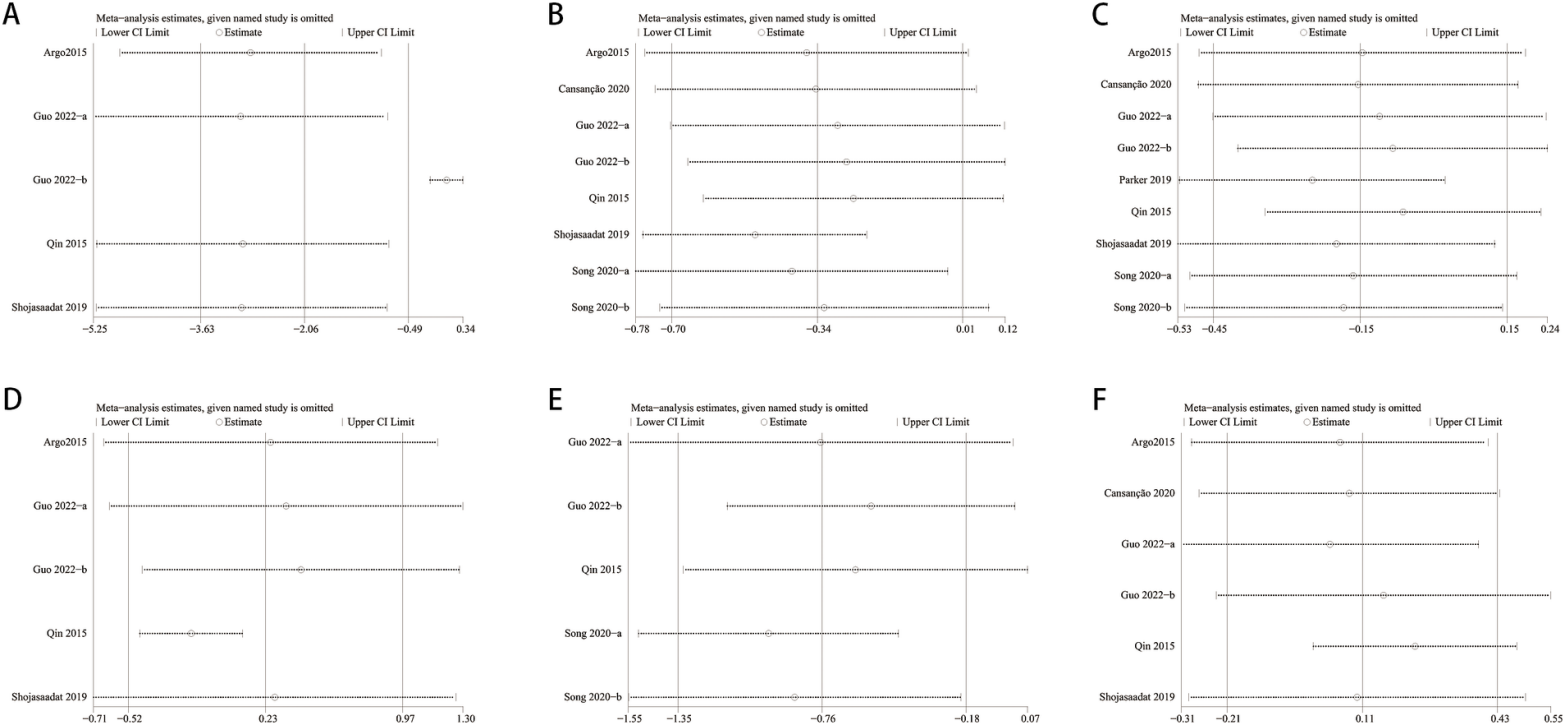
Sensitivity analysis of **(A)** HOMA-IR, **(B)** total cholesterol, **(C)** ALT, **(D)** insulin, **(E)** TNF-a and **(F)** FBS.

One-way sensitivity analyses were conducted to assess result stability and evaluate heterogeneity’s impact on the study outcomes. ALT, Total Cholesterol, insulin, TNF-*α*, and HOMA-IR were compared, and each study’s influence on the combined SMD was examined through sequential removal. Sensitivity analyses indicated consistent combined SMDs after excluding individual studies for FBS, ALT, and insulin. Total Cholesterol, TNF-α, and HOMA-IR showed significant fluctuations in the combined SMD, indicating result instability. Exclusion of the Shojasaadat-2019 and Song-2020 studies led to a shift from non-significant to significant findings for Total Cholesterol. Likewise, exclusion of the Qin-2015 and Guo-2022 studies reversed significant findings to non-significant for TNF-α. In terms of HOMA-IR, excluding the Guo-2022 study led to a transition from significant to non-significant outcomes (see Figure 12).

### 3.9 Subgroup analysis

Subgroup analyses were conducted based on the dosage of fish oil and the intervention duration to explore the stability of the results and potential sources of heterogeneity. For ALT, we found that there was a statistically significant difference when the intervention duration was greater than 12 weeks (*p* < 0.0001), while there was no statistical significance when it was less than 12 weeks (*p* = 0.1). The heterogeneity among subgroups decreased (I^2^ = 0). However, the dose subgroup analysis did not show statistical significance, and heterogeneity decreased in the subgroup less than 2000 mg (I^2^ = 36%). The subgroup analysis of AST indicated that there was statistical significance when the intervention dose was less than 2000 mg (*p* = 0.0006) and when the intervention duration was greater than 12 weeks (*p* = 0.0004); there was no statistical significance when the intervention dose was greater than 2000 mg (*p* = 0.45) and when the treatment duration was less than 12 weeks (*p* = 0.56). The subgroup analysis of GGT showed statistical significance when the intervention dose was less than 2000 mg (*p* = 0.02), but no statistical significance when it was greater than 2000 mg (*p* = 0.2). The subgroup analysis based on intervention duration did not reveal any statistical significance (see Table 2).

**Table 2:** Subgroup analysis.

Subgroup	ALT				AST				GGT			
	Study	SMD [95%CI]	*p* value	*I* ^2^	Study	SMD [95%CI]	*p* value	*I* ^2^	Study	SMD [95%CI]	*p* value	*I* ^2^
Total	9	−0.15 [−0.45, 0.15]	0.33	62%	8	−0.29 [−0.48, −0.10]	0.003	10%	8	−0.07 [−0.26, 0.12]	0.46	30%
Dose												
≥2000mg	5	−0.06 [−0.57, 0.44]	0.8	73%	4	-0.10 [−0.37, 0.17]	0.45	0%	4	0.18 [−0.09, 0.45]	0.2	0%
<2000mg	4	−0.27 [−0.61, 0.07]	0.12	38%	4	−0.46 [−0.72, −0.20]	0.0006	0%	4	−0.31 [−0.57, −0.04]	0.02	0%
Time of duration												
>12 weeks	5	−0.50 [−0.74, −0.26]	<0.0001	0%	4	−0.46 [−0.71, −0.20]	0.0004	0%	4	−0.24 [−0.50, 0.01]	0.06	0%
≤12 weeks	4	0.23 [−0.05, 0.51]	0.1	0%	4	−0.08 [−0.36, 0.20]	0.56	0%	4	0.14 [−0.14, 0.42]	0.33	16%

## 4 Discussion

Metabolic dysfunction-associated steatotic liver disease (MASLD) has become the leading cause of chronic liver disease globally, owing to the rising prevalence of obesity and its related metabolic syndrome, posing a significant public health issue (33). MASLD has a reported prevalence of approximately 30% in Western countries and ranges from 12 to 24% in Asia (34). MASLD comprises a spectrum of liver damage, ranging from simple steatosis to non-alcoholic steatohepatitis, fibrosis, cirrhosis, end-stage liver disease, and occasionally hepatocellular carcinoma. Advanced liver fibrosis is recognized as an independent risk factor for mortality (35). Additionally, MASLD patients are at increased risk of atherosclerosis and cardiovascular disease (CVD), which is the primary cause of death in this population (36). Prior evidence indicates an association among MASLD, insulin resistance, and type 2 diabetes (T2DM) (37, 38). Globally, 37.3% of T2DM patients are affected by MASLD (4). Furthermore, MASLD is associated with an increased risk of extrahepatic cancers, particularly colon cancer, gastric cancer, and certain hormone-related cancers, which pose the highest cancer risks (39). Over the past decade, a growing body of observational studies has revealed an association between MASLD and a heightened prevalence and incidence of chronic kidney disease (CKD) (40, 41). Hence, MASLD poses a considerable public health challenge. Lifestyle modifications remain the mainstay of therapy, proving effective in addressing metabolic syndrome, reducing hepatic fat accumulation, and halting disease progression in individuals. Nonetheless, their implementation and adherence may pose challenges. Various drugs, dietary supplements, and surgical interventions are being investigated and have shown effectiveness in managing MASLD. Our study provides an updated systematic review and meta-analysis of randomized controlled trials (RCTs) assessing the use of fish oil supplements in MASLD treatment.

The analysis results of this article demonstrate significant improvements or trends in liver enzymes, lipid profiles, and body measurements among participants who received fish oil supplementation. However, glycemic metabolism did not show improvement trends in these RCTs. Biochemical data on liver enzymes and metabolic status showed significant improvements in TG, AST, HOMA-IR, and waist circumference. Unlike findings from other meta-analyses, our observations suggest that fish oil supplement intake significantly reduces TNF-*α*, a crucial pro-inflammatory mediator. This reduction in pro-inflammatory mediators is linked to a decrease in low-grade chronic inflammation, providing favorable outcomes not only for Metabolic dysfunction-associated steatotic liver disease (MASLD) but also for cardiovascular health. Nevertheless, fish oil supplementation did not result in significant benefits for ALT, GGT, FBS, CK18-M30, BMI, HipC, WHR, HDL, LDL, adiponectin, Total Cholesterol, insulin, or UA. Supplementing with fish oil also has an impact on the imaging and biopsy scores of MASLD patients. There have been reports in the literature that after supplementing with fish oil, improvements in steatosis, fibrosis, lobular inflammation, ballooning, and liver fat percentage were confirmed through ultrasound, MR, and liver biopsy (42). However, due to the limited number of relevant studies included in this research, a comprehensive analysis was not possible, therefore, more research is needed in the future.

Variables showing high heterogeneity, including HOMA-IR, Total Cholesterol, ALT, Insulin, and TNF-*α*, underwent sensitivity analysis. The results indicated instability in HOMA-IR, Total Cholesterol, and TNF-α outcomes, with unclear sources of heterogeneity. Interpretation of these meta-analytical findings should be cautious due to potential confounders. The results of the subgroup analysis on ALT, AST, and GGT show that when the fish oil dosage is less than 2000 mg and the treatment duration is more than 12 weeks, the heterogeneity significantly decreases, indicating that the heterogeneity is mainly related to the dosage and treatment duration. At the same time, statistically, the optimal fish oil treatment dosage may be no more than 2000 mg, and the treatment duration should be more than 12 weeks. However, due to the limited number of studies included in this research, further studies may be needed. The literature included in this study did not analyze the side effects of fish oil, but some articles (43) indicate that the side effects of taking fish oil supplements are minimal and comparable to the control group.

The mechanism by which fish oil effectively alleviates MASLD can be summarized as follows: Fish oil may positively influence cell membrane fluidity. Increased membrane fluidity is positively associated with GLUT4 translocation to the cytoplasm (44, 45). Enhanced membrane fluidity can concurrently reinstate the tyrosine kinase activity of insulin receptor substrates (IRS)-1 and − 2, facilitating insulin signaling transduction (46, 47). Another potential mechanism contributing to the development of MASLD is chronic low-grade inflammation. Hepatic triglyceride accumulation is associated with macrophage recruitment, leading to the synthesis of pro-inflammatory cytokines such as TNF-*α*, IL-1β, and IL-6. Extensive evidence suggests that fish oil (FO) intervention inhibits the toll-like receptor (TLR)-4 signaling pathway (48, 49). Consequently, pro-inflammatory cytokine production is suppressed. Additionally, fish oil (FO) supplementation may ameliorate MASLD by inhibiting triglyceride (TG) synthesis and promoting TG oxidation. FO modulates various nuclear receptors (PPAR family) and transcription factors (SREBP) responsible for lipid synthesis and metabolism (50, 51). Moreover, polyunsaturated fatty acids (PUFA) regulate transcription factors that control the expression of proteins involved in *de novo* lipogenesis, such as acetyl-CoA carboxylase (Acc) and fatty acid synthase (Fasn) (52, 53). This process inhibits de novo lipogenesis, which is a major contributor to hepatic steatosis (54).

This study has several limitations. Firstly, heterogeneity among the RCTs and sensitivity analysis indicates instability in some results, with observed publication bias for certain indicators. Secondly, the overall sample size of the seven included RCTs is relatively small, we attempted to conduct a meta-regression, but due to the limited number of studies included, there was a significant imbalance in the results. Thirdly, variations exist in treatment doses, durations, and protocols, although a subgroup analysis was conducted, the results may be uncertain due to the insufficient number of studies included. Further research is necessary to establish the dose-effect relationship of fish oil in treating fatty liver disease. This article represents the first meta-analysis investigating MASLD treatment with fish oil. All included studies are RCTs, providing tightly controlled confounding factors and baseline levels, reflecting a high level of evidence. This analysis highlights the effectiveness of fish oil in treating MASLD, broadening clinical options.

## 5 Conclusion

Current evidence suggests that fish oil supplementation improves plasma levels of AST, TG, TNF-*α*, and HOMA-IR, as well as waist circumference in the treatment of MASLD. Further research requires large sample sizes and long-term follow-up in randomized controlled trials to confirm these benefits.

## Data Availability

The original contributions presented in the study are included in the article/Supplementary material, further inquiries can be directed to the corresponding author.
