# Supplementary material for: Efficacy of fish oil supplementation on metabolic dysfunction-associated steatotic liver disease: a meta-analysis
Source: Front Nutr. 2025 Jan 24;12:1524830. doi: 10.3389/fnut.2025.1524830 (PMC11804523; doi:10.3389/fnut.2025.1524830)
Supplement: Supplementary file 1 [file Table_1.docx]

| **Supplementary Table S1:Search Strategy** | | |
| --- | --- | --- |
| Database | Search Strategy | Result |
| Pubmed | **(("Fish Oils"[Mesh]) OR ((((((Oils, Fish) OR (Fish Oil)) OR (Oil, Fish)) OR (Fish Liver Oils)) OR (Liver Oils, Fish)) OR (Oils, Fish Liver))) AND (("Non-alcoholic Fatty Liver Disease"[Mesh]) OR (((((((((((((Non alcoholic Fatty Liver Disease) OR (NAFLD)) OR (Nonalcoholic Fatty Liver Disease)) OR (Fatty Liver, Nonalcoholic)) OR (Fatty Livers, Nonalcoholic)) OR (Liver, Nonalcoholic Fatty)) OR (Livers, Nonalcoholic Fatty)) OR (Nonalcoholic Fatty Liver)) OR (Nonalcoholic Fatty Livers)) OR (Nonalcoholic Steatohepatitis)) OR (Nonalcoholic Steatohepatitides)) OR (Steatohepatitides, Nonalcoholic)) OR (Steatohepatitis, Nonalcoholic)))** | 438 |
| **Web Of Science** | **(("Fish Oils"[Mesh]) OR ((((((Oils, Fish) OR (Fish Oil)) OR (Oil, Fish)) OR (Fish Liver Oils)) OR (Liver Oils, Fish)) OR (Oils, Fish Liver))) AND (("Non-alcoholic Fatty Liver Disease"[Mesh]) OR (((((((((((((Non alcoholic Fatty Liver Disease) OR (NAFLD)) OR (Nonalcoholic Fatty Liver Disease)) OR (Fatty Liver, Nonalcoholic)) OR (Fatty Livers, Nonalcoholic)) OR (Liver, Nonalcoholic Fatty)) OR (Livers, Nonalcoholic Fatty)) OR (Nonalcoholic Fatty Liver)) OR (Nonalcoholic Fatty Livers)) OR (Nonalcoholic Steatohepatitis)) OR (Nonalcoholic Steatohepatitides)) OR (Steatohepatitides, Nonalcoholic)) OR (Steatohepatitis, Nonalcoholic)))** | 191 |
| Cochrane Central Register of Controlled Trials | **((Fish Oils) OR ((((((Oils, Fish) OR (Fish Oil)) OR (Oil, Fish)) OR (Fish Liver Oils)) OR (Liver Oils, Fish)) OR (Oils, Fish Liver))) AND ((Non-alcoholic Fatty Liver Disease) OR (((((((((((((Non alcoholic Fatty Liver Disease) OR (NAFLD)) OR (Nonalcoholic Fatty Liver Disease)) OR (Fatty Liver, Nonalcoholic)) OR (Fatty Livers, Nonalcoholic)) OR (Liver, Nonalcoholic Fatty)) OR (Livers, Nonalcoholic Fatty)) OR (Nonalcoholic Fatty Liver)) OR (Nonalcoholic Fatty Livers)) OR (Nonalcoholic Steatohepatitis)) OR (Nonalcoholic Steatohepatitides)) OR (Steatohepatitides, Nonalcoholic)) OR (Steatohepatitis, Nonalcoholic))) (Topic)** | 32 |
| Embase | **((Fish Oils) OR ((((((Oils, Fish) OR (Fish Oil)) OR (Oil, Fish)) OR (Fish Liver Oils)) OR (Liver Oils, Fish)) OR (Oils, Fish Liver))) AND ((Non-alcoholic Fatty Liver Disease) OR (((((((((((((Non alcoholic Fatty Liver Disease) OR (NAFLD)) OR (Nonalcoholic Fatty Liver Disease)) OR (Fatty Liver, Nonalcoholic)) OR (Fatty Livers, Nonalcoholic)) OR (Liver, Nonalcoholic Fatty)) OR (Livers, Nonalcoholic Fatty)) OR (Nonalcoholic Fatty Liver)) OR (Nonalcoholic Fatty Livers)) OR (Nonalcoholic Steatohepatitis)) OR (Nonalcoholic Steatohepatitides)) OR (Steatohepatitides, Nonalcoholic)) OR (Steatohepatitis, Nonalcoholic))) (Topic)** | 230 |
