# Supplementary material for: Efficacy of fish oil supplementation on metabolic dysfunction-associated steatotic liver disease: a meta-analysis
Source: Front Nutr. 2025 Jan 24;12:1524830. doi: 10.3389/fnut.2025.1524830 (PMC11804523; doi:10.3389/fnut.2025.1524830)
Supplement: Supplementary file 2 [file Table_2.docx]

| Supplementary Table S2. Jadad quality scores of the trials included in the meta-analysis. | | | | |
| --- | --- | --- | --- | --- |
| Authors | Randomization method | Double blinding | Withdrawals/dropouts | \| Total \|  \| \| --- \| --- \| \|  \| |
|  |  |  |  |  |
| Argo2015 | 2 | 2 | 1 | 5 |
| Qin2015 | 1 | 2 | 1 | 4 |
| Parker 2019 | 2 | 2 | 1 | 5 |
| Shojasaadat2019 | 2 | 2 | 1 | 5 |
| Song2020 | 2 | 2 | 0 | 4 |
| Cansanção2020 | 2 | 2 | 1 | 5 |
| Guo2022 | 2 | 2 | 1 | 5 |
|  |  |  |  |  |
|  |  |  |  |  |
|  |  |  |  |  |
